# Dissipation and persistence behaviour of fipronil and its metabolites in chilli fruits using GC-ECD, confirmed by GC-MS, under semi-arid conditions

**DOI:** 10.1016/j.heliyon.2024.e39109

**Published:** 2024-10-11

**Authors:** Ramgopal Dudwal, B.L. Jakhar, A.R.K. Pathan, Alka Kataria, Gaurav Gupta, Vinoth Kumarasamy, Haider Ali, Kumud Pant

**Affiliations:** a School of Agriculture, Suresh Gyan Vihar University, Jaipur, Rajasthan, 302017, India; b Sri Karan Narendra Agriculture University, Jobner, Jaipur, Rajasthan, 303329, India; c IIS (Deemed to be University), Jaipur, 302020, India; d Centre for Global Health Research, Saveetha Medical College, Saveetha Institute of Medical and Technical Sciences, Saveetha University, India; e Centre for Research Impact & Outcome, Chitkara College of Pharmacy, Chitkara University, Rajpura, Punjab 140401, India; f Department of Parasitology and Medical Entomology, Faculty of Medicine, Universiti Kebangsaan Malaysia, Jalan Yaacob Latif, 56000, Cheras, Kuala Lumpur, Malaysia; g Department of Pharmacology, Kyrgyz State Medical College, Bishkek, Kyrgyzstan; h Graphic Era (Deemed to be University), Clement Town, 248002, Dehradun, India; i Research and Enterprise, University of Cyberjaya, Persiaran Bestari, Cyber 11, 63000 Cyberjaya, Selangor, Malaysia

**Keywords:** Dissipation, Persistence, Fipronil, Metabolites and Chilli

## Abstract

Chilli, one of the most popular vegetables in the world is infested by many insect-pests and diseases. Fipronil, a phenylpyrazole class insecticide is used to manage insect-pests on chilli. The present study aimed to analyze the dissipation patterns and residual concentrations of the fipronil and its metabolites in chilli fruits during the Kharif season of 2020–21, in semi-arid environment. In the study, fipronil was applied to the plants twice, with a 10-day gap between treatments, using a 5 % suspension concentrate. The applications were undertaken using two separate concentrations *i.e.* the lower dosage of 40 g a.i. ha^−1^ and higher dosage of 80 g a.i. ha^−1^. There were four sets of data for each concentration. The chilli crop was systematically sampled at predetermined intervals after the application of the second spray. The procedure encompassed utilizing the modified QuEChERS (Quick, Easy, Cheap, Effective, Rugged, and Safe) technique for extraction and purification, followed by analyzing the resulting residues using Gas Chromatography Electron Capture Detector. Gas Chromatography- Mass Spectrometry was subsequently conducted for the confirmation of the findings. The research revealed that the mean initial deposit of fipronil and its metabolites (desulfinyl, sulphide, and sulfone) at the authorized dosage was determined to be 0.574, 0.123, 0.031, and 0.180 mg kg^−1^, respectively. In contrast, when administered at twice the prescribed dosage, the mean first deposit was 1.204, 0.230, 0.067, and 0.382 mg kg^−1^. The half-life values of these residues exhibited a range of 1.2–4.1 days for both dosages. A prudent waiting duration was determined for the doses, leading to the conclusion that an average interval of 7 days is deemed safe for harvesting chilli peppers. The significance of this discovery is related to the maximum residue limits of 0.001 mg kg^−1^ for fipronil in green chilli, as established by the Food Safety and Standards Authority of India. This study provides significant insights into fipronil's persistence and proper management in chilli plant cultivation, emphasizing the importance of following prescribed dosages and designated waiting intervals to ensure the safety of food products.

## 1 Introduction

Now a days, various pesticides are extensively used on vegetable crops to combat insect infestations and meet the rising demand for vegetables amid a growing population. Modern agriculture has incorporated chemical fertilizers and pesticides, which are unique in that they are sprayed on crops specifically to fight pests, in an effort to boost productivity.The significant developments of the green and grey revolutions in plant agriculture, the yellow process in oilseed crops, and the blue and white revolutions in animal husbandry have been substantially supported by chemical pesticides. However, their continuous, excessive, and indiscriminate use poses risks of insecticide residues, which concernsboth farm laborers and consumers. To address this issue, precise analytical and decontamination methods are essential for detecting and controlling quantified residues at or below the maximum residue limits, ensuring safe consumption [1,2]. Many authors, including Dudwal et al., 2024 [3] have researched and proposed several decontamination methods to mitigate residues of insecticide residues.

Chemical pesticides, as biologically active compounds, play a crucial role globally in protecting food, fiber, and human health [[4], [5], [6], [7]]. Fipronil, a phenylpyrazole class insecticide, disrupts insect nerve function by targeting the γ-amino butyric acid type A (GABA) receptor system [8]. Fipronil degrades into significant metabolites in environmental settings through various processes [[9], [10], [11], [12]]. Among these, the desulfinyl photodegrade is notably stable and more toxic than fipronil, posing heightened toxicity to mammals and freshwater invertebrates [13,14]. Fipronil is labelled for use in numerous crops and is effective against various insect pests, including rice stem borer, leaf folder, and household pests [15,16]. Its widespread application, through various means such as soil, foliar, bait, or seed treatment, is essential in controlling soil and foliar insects in diverse crops like rice, vegetables, and fruits [9,[17], [18], [19]]. However, its degradation leaves residues in plants, water, and soil, impacting human and animal health, with effects ranging from acute symptoms like headaches and nausea to chronic impacts like cancer and reproductive harm [[20], [21], [22], [23]].

Chilli (*Capsicum annum* L.), a member of the Solanaceae family, is a widely consumed and commercially significant spice and vegetable crop known for its color, piquancy, and health benefits. India, as the top consumer and exporter, faces challenges in chilli production due to climatic conditions, seed quality, diseases, and pests, with significant yield loss attributed to thrips and mites [24,25].The increasing demand for pesticide-free foods has spurred research into regulatory standards for pesticide residues in foods. Maximum Residue Limits (MRL) set by regional authorities or the Codex Alimentarius are critical in reducing pesticide intake and ensuring consumer health. The Food Safety and Standards Authority of India, 2014 [26], have established an maximum residue limits of 0.001 mg kg^−1^ for fipronil on green chilli, highlighting the importance of adhering to these standards for global food safety. Nonetheless, inappropriate agronomic practices, such as excessive chemical pesticide use, can lead to high residue levels in soil and chilli. This can further affect beneficial microorganisms in the soil, which could reduce the nutritional value of chilli by decreasing the levels of certain nutrients, such as vitamins and minerals [27].

The initial deposition of pesticides is influenced by numerous factors, including concentration, weather conditions, and plant characteristics, while dissipation depends on crop stage, weather factors, and surface characteristics of the substrate [28]. This study used foliar sprays at both the recommended and double-recommended levels to further understand how fipronil and its metabolites break down and persist in chilli fruits in semi-arid environments in Rajasthan, India. The purpose is to sensitize about the repercussions of chemical pesticide residues on the health of living organisms.

## 2 Materials and methods

### 2.1 Chemicals and reagents

During the investigation, analytical grade solvents were utilized. Solvents such as acetone and *n*-hexane of analytical reagent grade, as well as acetonitrile of HPLC grade, were sourced from Merck, Darmstadt, Germany. Activated anhydrous magnesium sulphate and primary secondary amine (PSA) sorbents were acquired from Agilent. The Certified Reference Materials (CRMs) of fipronil and its metabolites, including desulfinyl, sulfide, and sulfone, were obtainedfrom Sigma Aldrich, India, for the standard reference.

### 2.2 Preparation of standard solution

To prepare the individual stock solutions with a concentration of 200 mg kg^−1^, 10 mg of fipronil and its metabolites (desulfinyl, sulfide, and sulfone) were each liquefied in *n*-hexane and subsequently reconstituted to the mark in a 25 mL volumetric flask. From these 200 mg kg^−1^ stock solutions, 10 mg kg^−1^ standard solutions of fipronil and its metabolites were independently prepared in separate 25 mL volumetric flasks. Working standard solutions with concentrations of 1.00, 0.75, 0.50, 0.25, 0.10, 0.05, 0.01, 0.005, and 0.001 mg kg^−1^ were then meticulously made ready through serial dilution of the 10 mg kg^−1^ standard solution. These concentrations were injected into the Gas Chromatography (GC) system to generate a calibration curve. Both the standard stock and working standard solutions were stored at a temperature of 4 °C to maintain their stability. This preparation and storage procedure ensures the accuracy and reliability of the solutions for subsequent analytical applications.

### 2.3 Instruments

The study employed a Gas Chromatography-2010 system (Shimadzu) with an Electron Capture Detector for residue analysis. The findings were subsequently verified using a Gas Chromatography-Mass Spectrometer-2010 (Shimadzu) for confirmation. The chromatographic separation utilized capillary columns DB-5, which were distinguished by their dimensions of 30 m in length, 0.25 mm in internal diameter, and a film thickness of 0.25 μm. The sample preparation process employed ultrapure Milli-Q water to achieve the utmost purity and reliability in the analytical method. In addition, a calibrated analytical balance was used to measure the weight of both samples and pesticide references correctly. The balance possessed a precise weighing range spanning from 0.001 to 100 g. Maintaining precise measurements was of utmost importance in upholding the integrity of the analytical process and guaranteeing the dependability of the results from the studies conducted using Gas Chromatography Mass Spectrometry (GCMS).

### 2.4 Field experiment

The field trial was conducted at the Horticulture Farm of the Rajasthan Agricultural Research Institute (RARI), Durgapura, Jaipur. The residue analyses were performed in the All India Network Project on Pesticide Residues (AINP-PR) Laboratory, Division of Entomology at Rajasthan Agricultural Research Institute (RARI), Durgapura, Jaipur. A RBD (Randomized Block Design) was utilized for plot organization, including four replications per treatment. Key details such as crop variety, treatment specifics, plot dimensions, planting spacing, and critical dates like sowing and transplanting are comprehensively detailed in Table 1.

**Table 1:** Details of experiment.

Crop	Chilli
Variety	Kranti
Treatments	3 (i). Lower dose *i.e.* 40 g a.i. ha^−1^ (ii). Higher dose *i.e.* 80 g a.i. ha^−1^ (iii). Control
Plot size	1.8 × 5.0 m
Spacing	60 × 45 cm
Date of sowing	July 2020
Date of transplanting	August 2020 *i.e.*after 30 days of sowing

### 2.5 Application of insecticide

The fipronil insecticide formulation was applied on the crop (Chilli) through a hand-operated knapsack sprayer. The pesticide was sprayed at two different concentrations *i.e.* the lower dose of 40 g a.i. ha^−1^ and higher dose of 80 g a.i. ha^−1^. The initial spraying was done at the start of fruit formation, followed by a second application ten days later, thus completing a two-spray regimen for the chilli crop. To minimize drift caused by heavy winds and ensure precise application, all spraying activities were conducted during the morning hours, ensuring the uniformity of treatment across different plots.

### 2.6 Sampling

Chilli fruit samples (1 kg) were collected from each replicate, from both treated and untreated plots. The collection intervals were set at 0 (2 h post-treatment), 1, 3, 5, 7, 10, 15, and 20 days following the 2nd spray. These samples were carefully packaged in polyethylene bags, labelled, and shipped off to the lab to thoroughly examine pesticide residue.

### 2.7 Extraction and cleanup

Chilli fruits were analyzed using the QuEChERS (Quick, Easy, Cheap, Effective, Rugged, and Safe) technique, recognized for their efficiency and effectiveness in residue analysis. Initially, each chilli fruit sample was finely chopped and thoroughly mixed to ensure homogeneity.Acetonitrile was added to remove the contaminants from the samples. Then, sodium chloride was used to this extract to reduce polar interferences. MgSO_4_ (Anhydrous magnesium sulphate) and PSA (Primary Secondary Amine) were also used for the final cleanup phase in a dispersive solid-phase extraction. An essential part of the extraction's success was the anhydrous magnesium sulphate's function in drying out the organic phase. Primary secondary amine (PSA) selectively remove compounds that could impact the results of the residue analysis, such as sugars, fatty acids, organic acids, lipids, and certain pigments. This process is outlined in the study conducted by Dudwal et al., 2023a [2].

A 15 g sample was extracted from a 1 kg batch of homogenized chilli and transferred into a centrifuge tube with a volume of 50 mL. A volume of 30 mL of acetonitrile was added to the mixture. Subsequently, the tube was hermetically sealed and subjected to vigorous agitation using a vortex mixer at high velocity for 1 min, ensuring thorough mixing of the solvent with the sample. Following this, the samples underwent further homogenization using a low-volume homogenizer operating at a speed of 14,000 to 15,000 rpm (equivalent to 24,104 to 27,670 Relative Centrifugal Force, RCF) for a duration of 2–3 min.Then, 3 g of sodium chloride was added into the mixture and shaken vigorously for 2 min. This step is crucial for facilitating the partitioning of the organic and aqueous phases. The mixture was then centrifuged for 3 min at a speed ranging between 2500 and 3000 rpm (769–1107 RCF) to separate the organic layer effectively.The next phase involved gently transferring around 18 mL of the organic layer into a 50 mL test tube. After that, 9 g of anhydrous sodium sulphate was added in the test tube. The addition was made to eliminate any leftover moisture in the sample. The test tube was then shaken vigorously to ensure that the anhydrous sodium sulphate made good contact with the organic layer, allowing for the most effective removal of any remaining moisture,as outlined in the study conducted by Dudwal et al., in 2023b [29].

In the second phase of the experimental protocol, 11 mL of the organic layer extract collected earlier was carefully placed into a centrifuge tube with a capacity of 15 mL. The tube was filled with a pre-mixed solution consisting of 0.4 g of primary secondary amine (PSA) and 1.15 g of anhydrous magnesium sulphate (MgSO_4_). The tube contents were further agitated extensively using a vortex mixer for 30 s, guaranteeing the homogeneous distribution of the solid-phase materials inside the extract. The tube underwent centrifugation for 5 min at a rotational speed range of 2500–3000 revolutions per minute (rpm), corresponding to a relative centrifugal force (RCF) range of 769–1107. This procedure enabled the subsequent segregation and elucidation of the amalgamation. Then, 6 mL of the cleared extract was carefully taken into a test tube. The solvents present in the extract were subsequently removed by evaporation using a Turbo Vap evaporator. The temperature during this process was carefully controlled to remain below 40 °C to avoid any potential degradation of the analytes. After drying, the remaining substance was dissolved in 3 mL of *n*-hexane inside the test tube, facilitating the dissolution of any remaining residues. This process is outlined in the study conducted by Dudwal et al., 2024 [3].

The final stage in sample preparation involved filtration of the reconstituted mixture using a syringe filter made of polytetrafluoroethylene (PTFE) with a pore size of 0.22 μm. The process of filtration was employed to effectively eliminate any particle debris, resulting in the production of a visually transparent solution. The solution that had undergone filtration was then carefully taken into a glass vial, preparing it for the examination of residues using Gas Chromatography with Electron Capture Detection (GC-ECD) and Gas Chromatography-Mass Spectrometry (GC-MS) techniques.

### 2.8 Method validation

The technique used in this study underwent thorough validation according to the SANATE guidelines [30]. The validation of analytical procedures is a crucial aspect of maintaining scientific rigour, as it guarantees the appropriateness of these methods for their intended purposes. The importance of conducting validations to ensure analytical data's correctness, precision, and dependability has been emphasized by Huber, 2007 [31]. The method includes a series of tests for method validation to ensure the accuracy and reliability of the analytical techniques used. The signal-to-noise ratio (S/N) was the key parameter used to determine the pesticide's Limits of Determination and Limits of Quantitation. With a signal-to-noise (S/N) ratio of 3:1, the concentration of an analyte below which a positive result may be reliably identified is known as the Limits of Determination. In contrast, Limits of Quantitation was established by measuring artificially enriched samples at a spiking concentration of 0.001 mg kg^−1^ with a signal-to-noise (S/N) ratio of 10:1, as outlined in the study conducted by Dudwal et al., 2023b [29].

A matrix-matching calibration curve was plotted to evaluate the method's linearity. The curve plotted using nine concentrations (ranging from 0.001 to 1.0 mg kg^−1^) against their respective detector responses. Further, recovery experiments conducted on spiked samples evaluated the method's accuracy. These recovery tests were performed in quadruplicate, fortifying the samples with a pesticide mixture of 0.01, 0.005, and 0.001 mg kg^−1^.

The recovery percentage was determined by the given formula [2]:

$$\text{Recovery}\;\text{Percentage}=\frac{Sample\;peak\;area}{Standard\;peak\;area}\times 100$$

### 2.9 Analysis of fipronil and its metabolites residues

Fipronil and its metabolites were tested using a Gas Chromatography system equipped with an Electron Capture Detector (Model 2010, Shimadzu, Japan). Samples were injected in split mode into the Gas Chromatography Electron Capture Detector for pesticide residue detection and quantification. Residue identification was based on retention time comparisons with known standards under identical conditions. Critical parameters such as injector and detector temperatures, column flow, injection volume, split ratio, and retention times are detailed in Table 2.

**Table 2:** The functioning criteria of Gas Chromatography.

Column **temperature**^**°**^**C**	**Rate (**^**°**^**C/minute)**	**Temperature (**^**°**^**C)**	**Hold time (minutes)**
	–	160.0	1.00
	7.0	280.0	5.00
Injector temperature ^**°**^**C**	280		
Temperature of detector ^**°**^**C**	300		
**Gas flow rate (ml min**^**−**^**^1^)**			
Total flow (Detector)		12.0	
Flow of Column		1.50	
Aliquot injected		1 μl	
Split ratio		5	
**Retention time of fipronil and its metabolites**			
Fipronil		8.519 min	
Fipronil metabolite I = MB 046513		6.604 min	
Fipronil metabolite II = MB 046136		9.973 min	
Fipronil metabolite III = MB 045950		8.279 min	

The following formula was used to compute the residue quantities on a peak area basis [29].

$$\text{Residues}\;\left(\mu g/g\right)=\frac{\text{Peak}\;\text{area}\;\left(\text{Sample}\right)\;x\;\text{Conc}.\;\text{std}\;\left(\text{ppm}\right)\;x\;\mu \text{Lstd}\;\text{injected}\;x\;\text{Final}\;\text{volume}\;\text{of}\;\text{the}\;\text{sample}\;\left(1.5\;\text{mL}\right)}{\text{Peak}\;\text{area}\;\left(\text{Std}\right)\;x\;\text{weight}\;\text{of}\;\text{the}\;\text{sample}\;\left(1.5\;g\right)\;x\;\mu L\;\text{of}\;\text{sample}\;\text{injected}}$$

$$\text{Wt}.\;\text{of}\;\text{sample}\;\left(g\right)=\frac{\text{Sample}\;\text{wt}.\;\left(15\;g\right)\;x\;\text{aliquot}\;\text{taken}\;\left(3\;\text{mL}\right)}{\text{Volume}\;\text{of}\;\text{acetonitrile}\;\left(30\;\text{ml}\right)}=1.5\;g$$

#### 2.9.1 Residues dissipation rate

The following formula [29] was used to determine the percentage of residue that had dissipated relative to the initial deposit, for different time intervals.

$$\text{Percent}\;\text{dissipation}=\frac{\text{Initial}\;\text{deposit}\;–\;\text{Residues}\;\text{at}\;\text{given}\;\text{time}}{\text{Initial}\;\text{deposit}}\;x\;100$$

#### 2.9.2 Residue half-life (RL_50_) values

The values of RL_50_ were computed according to the methodology outlined by Koppel, 1969 [32].

$$\text{RL}\_\left(50\right)=\frac{\log\;2}{ǀkǀ}$$

where ǀkǀ is the step of regression equation.

#### 2.9.3 Waiting period (safe)

The waiting period, denoted as T_tol_, is the designated duration required for the insecticide to elapse before it reaches the established tolerance level.

The waiting time calculation was conducted per the criteria provided by the Codex Alimentarius Commission (CAC) and the Food Safety and Standards Authority of India, 2014. The formula used for this calculation is as follows:

$$T_{\text{tol}\;}=\frac{\left[a-\mathrm{Log}\;\text{tol}\;\right]}{B}$$

Where.

T_tol_ = minimum time (in days) required for the pesticide residue to reach below the tolerance limit

a = log of the apparent initial deposits obtained in the regression equation (Y = a + b X)

tol = tolerance limit of the insecticide (MRL)

b = slope of the regression line.

### 2.10 Confirmation of residues

Fipronil and its metabolite residues were confirmed by utilizing a Gas Chromatography-Mass Spectrometry (GC-MS) technique, specifically employing the GC-MS-QP 2010 Plus instrument. The analysis used a Rxi-1ms capillary column with dimensions of 30m x 0.25 i.d. x 0.25 μm. Before analyzing fipronil and its metabolites, the instrument was calibrated to ensure its accuracy, and all relevant parameters were adjusted to appropriate levels. The detection method relied on the mass-to-charge (*m*/*z*) ratio analysis, wherein the observation of precursor and product ions was conducted in multiple reaction monitoring modes. The injector temperature was maintained at 260 °C in splitless mode, employing helium (99.99 %) as the carrier gas with a 1 mL/min flow rate.

### 2.11 Statistical analysis

The data was statistically analyzed using Microsoft Excel 2016. The trials were replicated four times, and the outcome of fipronil and its metabolites were reported as the mean value plus or minus the standard deviation.

## 3 Results and discussion

### 3.1 Extraction and cleanup

The QuEChERS (Quick, Easy, Cheap, Effective, Rugged, and Safe) extraction and cleanup technique, widely recognized for its efficiency, has become a standard method for analyzing pesticide residues in food matrices, as noted by Anastassiades et al., 2003 [33] and Lehotay et al., 2010 [34]. Its popularity stems from its lower solvent and sample volume requirements, reduced time consumption, and enhanced analytical performance. This study employed a QuEChERS-based extraction and dispersive solid-phase extraction (d-SPE) cleanup using primary secondary amine (PSA) and MgSO_4_, coupled with analysis on GC-ECD and GC-MS. This approach was selected to minimize matrix effects and optimize pesticide recovery.Commonly, in QuEChERS methodology, acetonitrile is the solvent of choice. However, due to its incompatibility with GC analysis – where it can increase the inner force of the chromatographic system – acetonitrile was substituted with hexane, which is more suitable with the requirements of this analytical technique.

### 3.2 Method validation

**Fig. 1:**
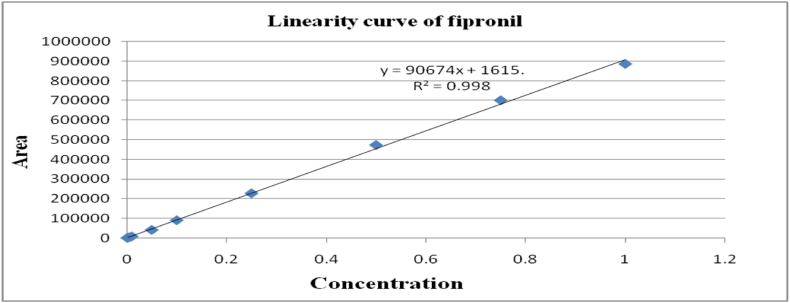
A standard curve of fipronil.

**Fig. 2:**
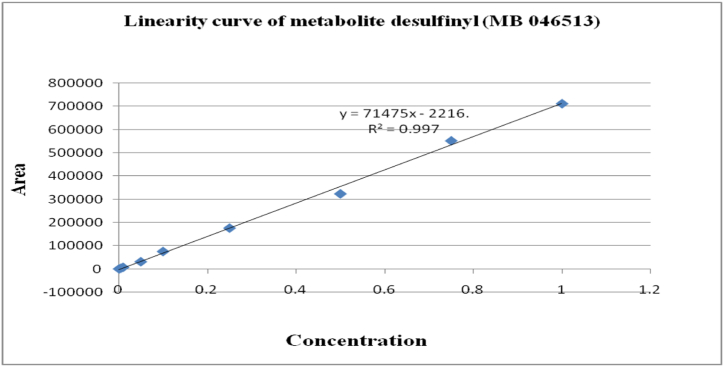
A standard curve of fipronil metabolite desulfinyl (MB 046513).

**Fig. 3:**
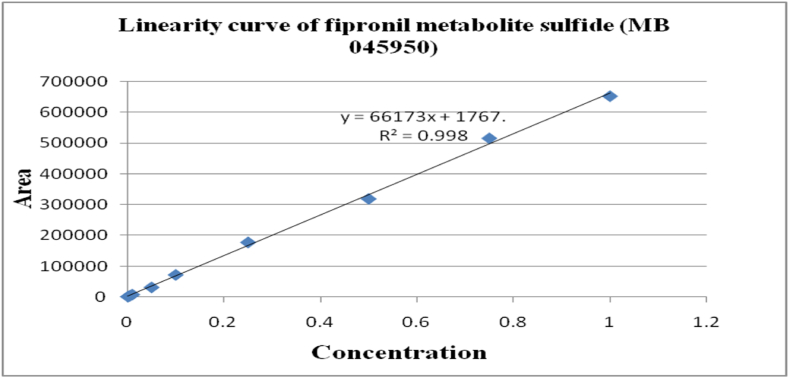
A standard curve of fipronil metabolite sulfide (MB 045950).

**Fig. 4:**
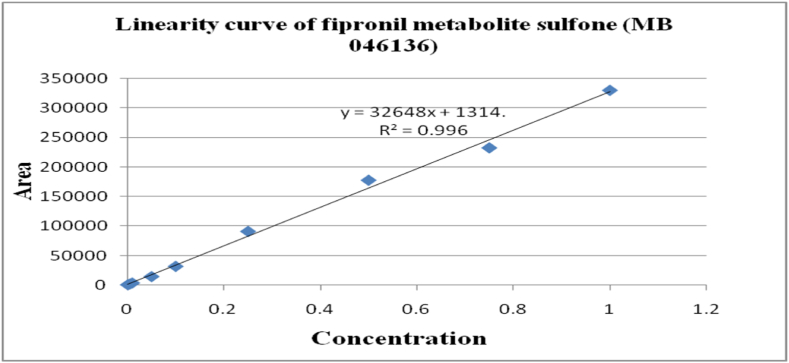
A standard curve of fipronil metabolite sulfone (MB 046136).

The method's validation comprised performance criteria, including recovery percentage, linearity, accuracy, sensitivity [LOD (Limits of Determination) and LOQ (Limits of Quantitation)], and precision in spiked samples. Matrix-matched calibration curves for chilli extracts demonstrated excellent linearity with correlation coefficients (R^2^) exceeding 0.99 for fipronil and its metabolites, as depicted in Fig. 1, Fig. 2, Fig. 3, Fig. 4. The determined LOQ (Limits of Quantitation) and LOD (Limits of Determination) were 0.001 mg kg^−1^ and 0.0003 mg kg^−1^ for fipronil and its metabolites (desulfinyl, sulfide, and sulfone). Recovery studies for these compounds in chilli fruits were also conducted to affirm result reliability. Chilli fruits' recoveries at three fortification levels (0.001, 0.005, and 0.01 mg kg^−1^) ranged from 87.3 to 98.0 %, as shown in Table 3, Table 4, aligning with SANTE 2020 standards.

**Table 3:** Percent recovery of fipronil and metabolite desulfinyl (MB046513) in chilli fruits at different fortification levels.

**Fortification level (mg kg-1)**	**Replications**	**Fipronil**		**Desulfinyl (MB046513)**	
		**μg recovered**	**Recovery (%)**	**μg recovered**	**Recovery (%)**
0.001	R_1_	0.00092	92.0	0.00089	89.0
	R_2_	0.00087	87.0	0.00088	88.0
	R_3_	0.00089	89.0	0.00091	91.0
	R_4_	0.00085	85.0	0.00093	93.0
**Mean ± SD**	**88.3 ± 2.586**			**90.3 ± 1.920**	
0.005	R_1_	0.0044	88.0	0.0043	86.0
	R_2_	0.0045	90.0	0.0047	94.4
	R_3_	0.0047	94.0	0.0046	92.0
	R_4_	0.0049	98.0	0.0049	98.0
**Mean ± SD**	**92.5 ± 3.841**			**92.6 ± 4.368**	
0.01	R_1_	0.00953	95.3	0.00895	89.5
	R_2_	0.00941	94.1	0.00917	91.7
	R_3_	0.00896	89.6	0.00875	87.5
	R_4_	0.00985	98.5	0.00927	92.7
**Mean ± SD**		**94.4 ± 3.192**		**90.4 ± 2.012**

**Table 4:** Percent recovery of fipronil metabolite sulfide (MB045950) and sulfone (MB046136) in chilli fruits at different fortification levels.

**Fortification level (mg kg-1)**	**Replications**	**Sulfide (MB045950)**		**Sulfone (MB046136)**	
		**μg recovered**	**Recovery (%)**	**μg recovered**	**Recovery(%)**
0.001	R_1_	0.00088	88.0	0.00086	86.0
	R_2_	0.00092	92.0	0.00090	90.0
	R_3_	0.00086	86.0	0.00088	88.0
	R_4_	0.00087	87.0	0.00085	85.0
**Mean ± SD**		**88.3 ± 2.278**		**87.3 ± 1.920**	
0.005	R_1_	0.0045	90.0	0.0051	102.0
	R_2_	0.0043	86.0	0.0049	98.0
	R_3_	0.0049	98.0	0.0047	94.0
	R_4_	0.0047	94.0	0.0049	98.0
**Mean ± SD**		**92.0 ± 4.472**		**98.0 ± 2.828**	
0.01	R_1_	0.00891	89.1	0.00987	98.7
	R_2_	0.00941	94.1	0.00961	96.1
	R_3_	0.00895	89.0	0.00924	92.4
	R_4_	0.00917	91.7	0.00897	89.7
**Mean ± SD**		**91.0 ± 2.104**		**94.2 ± 3.440**	

These findings are consistent with previous literature like Ann and ZehnderJarropp, 2016 [35], who has reported an R^2^ (correlation coefficients) value of 0.9647, Wu et al., 2017 [36] reported 0.9822 ± 0.9981, and Baldaniya et al., 2020 [37] reported R^2^ (correlation coefficients) values ≥ 0.99 for fipronil and its metabolites. Saini and Kumari, 2015 [38] reported LOD (Limits of Determination) of 0.0003 mg kg^−1^ and LOQ (Limits of Quantitation) of 0.001 mg kg^−1^, Aruna et al., 2015 [39] reported LOD (Limits of Determination) of 0.001 mg kg^−1^ and LOQ (Limits of Quantitation) of 0.005 mg kg^−1^, Ann and ZehnderJarropp, 2016(35) reported an LOQ (Limits of Quantitation) of 0.01 mg kg^−1^, and Wu et al., 2017 [36] reported an LOQ (Limits of Quantitation) of 0.01 mg kg^−1^. Other researchers, including Mohapatra et al., 2010; Chopra et al., 2010; Saini and Kumari, 2015; Aruna et al., 2015; Ann and ZehnderJarropp, 2016 and Wu et al., 2017 [35,36,[38], [39], [40], [41]] observed fipronil and its metabolite recoveries ranging from 80 to 93.0 % in various crops like grape leaves, berries, cotton lint, seeds, chilli, citrus, dried pepper berries, and cotton plants.

### 3.3 Analysis of residues, dissipation rates, half life and safe waiting period of fipronil and its metabolites

The tables (Table 5, Table 6) provided in this study present the residue levels of fipronil and its metabolites (desulfinyl, sulphide, and sulfone) in chilli fruits after the second application of the fipronil formulation. In untreated control samples of the chilli fruits, no traces of fipronil or its metabolites were detected. The investigation documented the mean initial concentrations of fipronil and its metabolites as 0.574, 0.123, 0.031, and 0.180 mg kg^−1^ at the prescribed dosage. In contrast, when administered at a dosage twice the prescribed amount, the mean initial concentrations were seen to be 1.204, 0.230, 0.067, and 0.382 mg kg^−1^, as assessed 2 h post-treatment. In addition, it was observed that the levels of residues present on chilli fruits were determined to be lower than the Limits of Quantitation (LOQ) of 0.001 mg kg^−1^ on the 15th, 10th, 7th, and 10th day when the prescribed dose of fipronil and its metabolites (namely, desulfinyl, sulphide, and sulfone) was applied. Similarly, on 20th, 15th, 10th, and 15th day, the residues were below the LOQ (Limits of Quantitation) when the dose was doubled.

**Table 5:** Residues (mg kg^−1^) of fipronil and its metabolites in chilli fruits at the lower dose (40 g a.i. ha^−1^).

**Days**	**Control**	**Fipronil**	**Desulfinyl (MB046513)**	**Sulfide (MB045950)**	**Sulfone (MB046136)**	**Total**
		**Mean**^a^**± SD**	**Mean**^b^**± SD**	**Mean**^b^**± SD**	**Mean**^b^**± SD**	
0	ND	0.574 ± 0.002 (0.0)^a^	0.123 ± 0.002 (0.0)	0.031 ± 0.002 (0.0)	0.180 ± 0.004 (0.0)	0.908 (0.0)
1	ND	0.380 ± 0.002 (33.8)	0.101 ± 0.002 (17.9)	0.019 ± 0.003 (38.7)	0.137 ± 0.003 (23.9)	0.637 (29.8)
3	ND	0.238 ± 0.016 (58.5)	0.074 ± 0.003 (39.8)	0.006 ± 0.001 (80.6)	0.066 ± 0.003 (63.3)	0.384 (57.7)
5	ND	0.182 ± 0.003 (68.3)	0.044 ± 0.002 (64.2)	0.002 ± 0.001 (93.5)	0.020 ± 0.001 (88.9)	0.248 (72.7)
7	ND	0.064 ± 0.003 (88.9)	0.015 ± 0.003 (87.8)	BDL	0.006 ± 0.001 (96.7)	0.085 (90.6)
10	ND	0.023 ± 0.004 (96.1)	BDL	–	BDL	0.023 (97.5)
15	ND	BDL	–	–	–	BDL
Correlation Coefficient R^2^ = 0.974			Correlation Coefficient R^2^ = 0.937	Correlation Coefficient R^2^ = 0.998	Correlation Coefficient R^2^ = 0.985 Regression Equation y = -0.212x + 1.348 RL_50_ = 2.2 Waiting Period (Days) = 5	
Regression eq. Log Y = −0.136x + 1.780			Regression Equation y = −0.123x + 1.156	Regression Equation y = -0.232x + 0.498		
RL_50_ = 3.4			RL_50_ = 3.8	RL_50_ = 1.2		
Waiting Period (Days) = 7			Waiting Period (Days) = 5	Waiting Period (Days) = 3		

a % reduction of four replications.

b Mean of four replications BDL = Below Determination Level ND=Not Detected.

**Table 6:** Residues (mg kg^−1^) of fipronil and its metabolites in chilli fruits at higher dose (80 g a.i. ha^−1^).

Days	Control	Fipronil	Desulfinyl (MB046513)	Sulfide (MB045950)	Sulfone (MB046136)	Total
		Mean∗∗ ± SD	Mean∗∗ ± SD	Mean∗∗ ± SD	Mea∗∗ ± SD	
0	ND	1.204 ± 0.017 (0.0)∗	0.230 ± 0.002 (0.0)	0.067 ± 0.004 (0.0)	0.382 ± 0.026 (0.0)	1.883 (0.0)
1	ND	1.085 ± 0.003 (9.9)	0.205 ± 0.003 (10.9)	0.043 ± 0.004 (35.8)	0.270 ± 0.015 (29.3)	1.603 (14.9)
3	ND	0.748 ± 0.003 (37.9)	0.120 ± 0.003 (47.9)	0.016 ± 0.001 (76.1)	0.137 ± 0.005 (64.1)	1.021 (45.8)
5	ND	0.323 ± 0.003 (73.2)	0.087 ± 0.003 (62.2)	0.006 ± 0.001 (91.0)	0.044 ± 0.008 (88.5)	0.460 (75.6)
7	ND	0.124 ± 0.003 (89.7)	0.033 ± 0.003 (85.7)	0.002 ± 0.001 (97.0)	0.015 ± 0.003 (96.1)	0.174 (90.8)
10	ND	0.073 ± 0.003 (93.9)	0.020 ± 0.003 (91.3)	BDL	0.006 ± 0.001 (98.4)	0.099 (94.7)
15	ND	0.015 ± 0.003 (98.8)	BDL	–	BDL	0.015 (99.2)
20	ND	BDL	–	–	–	BDL
Correlation Coefficient R^2^ = 0.987			Correlation Coefficient R^2^ = 0.977	Correlation Coefficient R^2^ = 0.996	Correlation Coefficient R^2^ = 0.988	
Regression eq. Log Y = −0.133x + 2.156			Regression Equation y = −0.113x + 1.406	Regression Equation y = −0.236x + 0.875	Regression Equation y = -0.189x + 1.617	
RL_50_ = 3.2			RL_50_ = 4.1	RL_50_ = 2.0	RL_50_ = 2.5	
Waiting Period (Days) = 10			Waiting Period (Days) = 7	Waiting Period (Days) = 5	Waiting Period (Days) = 7	

∗Per cent reduction of four replications.

∗∗Mean of four replications BDL = Below Determination Level ND=Not Detected.

**Fig. 5:**
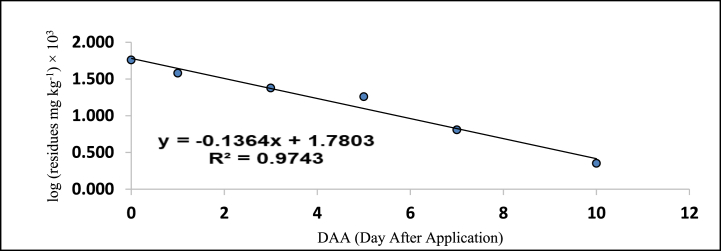
Dissipation kinetics of fipronil in chilli fruits at lower dose.

**Fig. 6:**
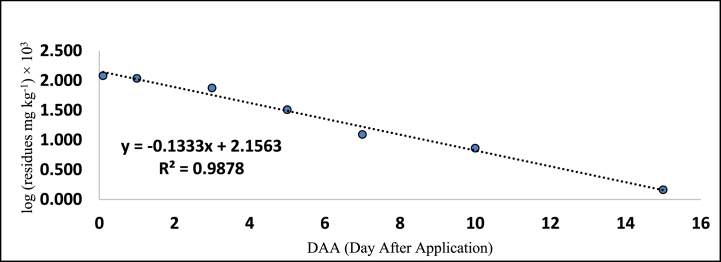
Dissipation kinetics of fipronil in chilli fruits at higher dose.

**Fig. 7:**
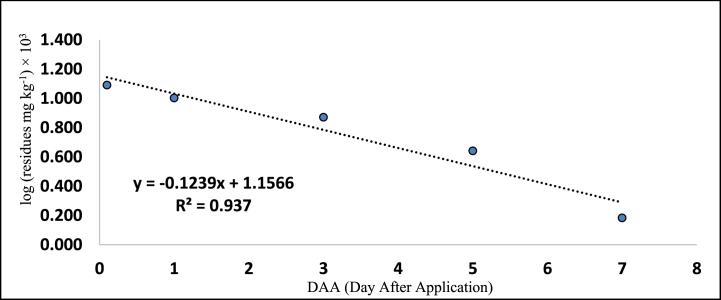
Dissipation kinetics of fipronildesulfinyl in chilli fruits at lower dose.

**Fig. 8:**
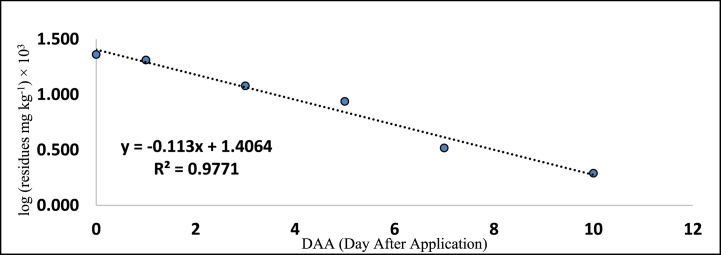
Dissipation kinetics of fipronildesulfinyl in chilli fruits at higher dose.

**Fig. 9:**
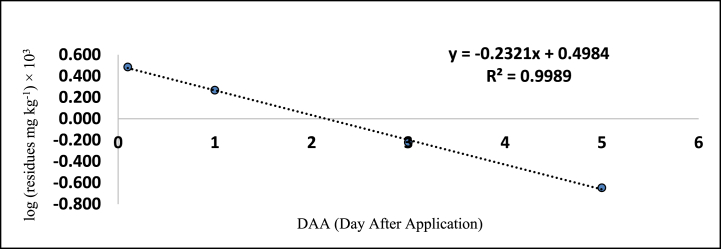
Dissipation kinetics of fipronil sulfide in chilli fruits at lower dose.

**Fig. 10:**
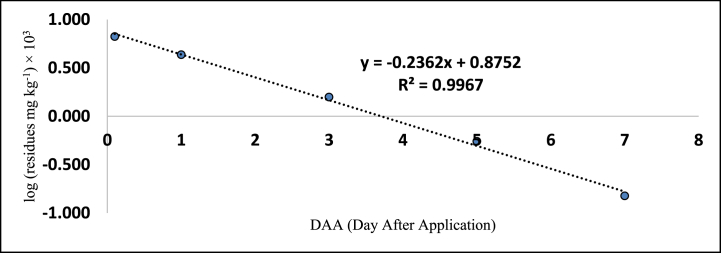
Dissipation kinetics of fipronil sulfide in chilli fruits at higher dose.

**Fig. 11:**
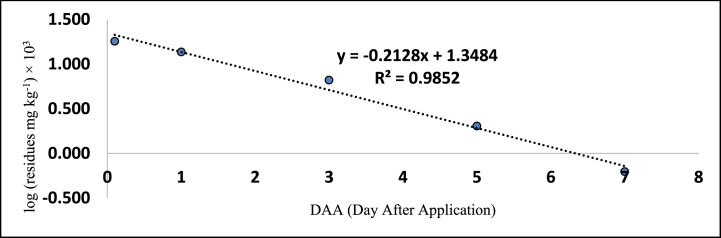
Dissipation kinetics of fipronil sulfone in chilli fruits at lower dose.

**Fig. 12:**
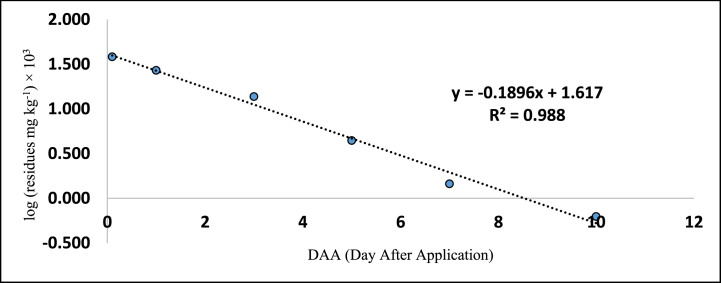
Dissipation kinetics of fipronil sulfone in chilli fruits at higher dose.

Table 5, Table 6 also provides the regression equations and correlation coefficients of fipronil and its metabolites in chilli fruits at the specified dosage levels. Furthermore, graphs depicting the relationship between the logarithm of residue concentration (expressed in mg kg^−1^, multiplied by 10^3^) and the duration of time (measured in days) were generated to analyze the degradation patterns (kinetics) of fipronil and its metabolites in chilli fruits. These graphs are presented in Fig. 5, Fig. 6, Fig. 7, Fig. 8, Fig. 9, Fig. 10, Fig. 11, Fig. 12. The values for residual half-life (RL_50_) were determined to be 3.4, 3.8, 1.2, and 2.2 days at the lower dose of 40 g a.i. ha^−1^, and 3.2, 4.1, 2.0, and 2.5 days at higher dose of 80 g a.i. ha^−1^, respectively. As a result, the duration of time required for the residues of fipronil and its metabolites (namely, desulfinyl, sulphide, and sulfone) to dissipate from chilli fruits was determined to be 7, 5, 3, and 5 days for the acceptable dosage, and 10, 7, 5, and 7 days for higher dosage, respectively. This observation indicates that under the climatic context of Rajasthan, the presence of fipronil residues and its metabolites does not exhibit prolonged persistence after the final application.

The findings of this study align with the conclusions reported in prior academic literature. Aruna et al., 2015 [39] investigated the lifespan of fipronil and its metabolites in citrus fruits in their study. The researchers observed the initial concentrations of fipronil, as 0.31 mg kg^−1^ and 0.59 mg kg^−1^ for the standard and double-recommended doses, respectively. The observed concentrations of metabolites were 0.41 mg kg^−1^ and 0.75 mg kg^−1^, respectively. Following the final treatment, the concentrations fell below the determination level (BDL) of 0.005 mg kg^−1^ on the 15th and 20th day. Another study by Hingmire et al., 2015 [42] revealed that the evaporation process of fipronil on okra had a half-life of 2.5 days. The researchers assessed the pre-harvest intervals (PHI) for single and double dosages of fipronil to be 15 and 19.5 days, respectively. Saini and Kumari, 2015 [38] conducted a study that specifically examined the enduring presence of fipronil and its metabolites in chilli. They measured the initial deposition concentrations of total fipronil (comprising fipronil and its metabolites) and found 0.409 mg kg^−1^ and 0.808 mg kg^−1^deposition for single and double doses, respectively. The major metabolite discovered by them, among the metabolites was desulfinyl, followed by sulfone and sulphide. The investigation revealed that the half-life of whole fipronil, when administered at both single and double dosages, was seen to be 3.50 days and 3.53 days. The findings of this study are consistent with the results of the current study, suggesting that a waiting period of 7 days for single doses and 10 days for double doses is necessary to ensure the safe use of chilli. Ann and ZehnderJarropp, 2016 [35] conducted a study to investigate the dissipation of fipronil and its three metabolites in black pepper. The results indicated that fipronil was identified as the predominant parent compound, whereas the sulfone derivative was observed to be the most prevalent metabolite. The metabolites of sulphide, amide, and desulfinyl were also seen. However, their quantities were below the determination level of 0.01 mg kg^−1^. The pre-harvest interval for applying Regent 80 WG was determined to be 12 days. Another experiment was conducted by Reddy et al., 2021 [43]. They sprayed fipronil at 500 g a.i.ha^−1^ and collected samples at 0, 1,3, 5, 7, 10 and 15 days after third spray. They found from their study that the initial deposits of 1.47 mg kg^−1^ were dissipated to below determination levels (BDL) on 15th day after third spray on chilli. The residues recorded were 0.97, 0.52, 0.41, 0.36 and 0.16 mg kg^−1^ at 1, 3, 5, 7, and 10 days after thirdapplication of fipronil, respectively. Mukherjee et al., 2021 [44] studied the dissipation behavior of fipronil and its metabolites in a paddy ecosystem. They found that about 92–96 % of fipronil residues degraded after 15 days of fipronil spraying, with a half-life of 3.4–4.1 days. At the time of harvesting of plant, the residues were foundto be below the level of quantification (<0.005 μg g^−1^).

### 3.4 Confirmation of residues

The identification of fipronil and its metabolites was achieved by employing gas chromatography-mass spectrometry (GC-MS) analysis. The analysis was performed using the Shimadzu GCMS-QP 2010 Plus equipment, equipped with a capillary column of 30 m in length, 0.25 mm in inner diameter, and coated with a 0.25 μm film of Rxi-1 ms. The experimental methodology encompassed examining a solution containing fipronil and its metabolites (desulfinyl, sulphide, and sulfone) at a predetermined concentration of 1 mg kg^−1^. The investigation was performed utilizing a scan mode, encompassing a mass-to-charge ratio (*m*/*z*) range of 213–368. The application of multiple reaction monitoring mode was employed to examine both fipronil and its metabolites. The injector temperature was maintained at a constant value of 260 °C, and helium was employed as the carrier gas at a flow rate of 1 mL/min. The molecular weights and fragment information for each molecule were as follows: fipronil desulfinyl (389.14, with fragments at 368, 281, and 231), fipronil sulphide (421.10, with fragments at 351, 228, and 213), fipronil sulfone (453.1, with fragments at 355, 255, and 213), and fipronil parent (437.1, with fragments at 351 and 367).

## 4 Conclusion

A controlled field experiment was conducted in semi-arid conditions to investigate fipronil's dissipation and persistence kinetics and its metabolites on chilli fruits. The trial utilized the Multilocational Good Agriculture Practice methodology. Fipronil is classified as a novel insecticide within the phenyl pyrazole category. The subject in question demonstrates various favourable characteristics, including a unique mode of operation that is non-harmful to pollinators and the surrounding ecological system. Due to its extended insecticidal activity, the chemical effectively manages thrips and moths on chilli plants.The findings of this study demonstrated a high level of accuracy and precision, particularly in the context of the dissipation kinetics of fipronil in chilli. As a result, it has been established that an average waiting period of 7 days is safe for harvesting chilli after treatment with fipronil. These current findings offer valuable insights into the safe use of pesticides on chilli crops to combat various insect pests and contribute to the establishment of maximum residue limits (MRL) and safe waiting periods for fipronil pesticides. This study holds significant importance for ensuring the safety of human consumption of chilli fruits when pesticides are employed as a protective measure.

## CRediT authorship contribution statement

**Ramgopal Dudwal:** Writing – original draft, Formal analysis, Conceptualization. **B.L. Jakhar:** Supervision. **A.R.K. Pathan:** Supervision. **Alka Kataria:** Writing – review & editing. **Gaurav Gupta:** Resources, Conceptualization. **Vinoth Kumarasamy:** Writing – review & editing, Funding acquisition. **Haider Ali:** Conceptualization, Resources. **Kumud Pant:** Formal analysis, Resources.

## Availability of data and materials

The data utilized in this study were obtained through a field experiment done by our research team. Detailed descriptions of the procedures employed are found in the publication.

## Data availability statement

Data will be made available on request.

## Declaration of competing interest

The authors declare that they have no known competing financial interests or personal relationships that could have appeared to influence the work reported in this paper.
